# Agile Application of Digital Health Interventions during the COVID-19 Refugee Response

**DOI:** 10.5334/aogh.2995

**Published:** 2020-10-15

**Authors:** Nirmala P. Narla, Aral Surmeli, Sean M. Kivlehan

**Affiliations:** 1 Division of Medical Critical Care, Boston Children’s Hospital and Harvard Medical School, MA, US; 2 Medical Rescue Association of Turkey (MEDAK), Istanbul, TR; 3 Department of Emergency Medicine, Brigham and Women’s Hospital and Harvard Medical School, Boston, MA, US; 4 Harvard Humanitarian Initiative, Cambridge, MA, US

## Abstract

The intersection of digital health platforms and refugee health in the context of the novel 2019 coronavirus disease (COVID-19) has not yet been explored. We discuss the ability of a novel mobile health (mhealth) platform to be effectively adapted to improve health access for vulnerable displaced populations. In a preliminary analysis of 200 Syrian refugee women, we found positive user feedback and uptake of an mhealth application to increase access to preventive maternal and child health services for Syrian refugees under temporary protection in Turkey. Rapid adaptation of this application was successfully implemented during a global pandemic state to perform symptomatic assessment, disseminate health education, and bolster national prevention efforts. We propose that mhealth interventions can provide an innovative, cost-effective, and user-friendly approach to access the dynamic needs of refugees and other displaced populations, particularly during an emerging infectious disease outbreak.

## Introduction

It is estimated that at least 200 million people will be forcibly displaced from their homes by 2050, driven by humanitarian crises often fueled by conflict and climate change [1]. In Syria, the Civil War has forcibly displaced twelve mill1ion people from their homes since 2011 [2]. Nearly four million live in Turkey, which hosts the largest Syrian refugee population in the world [2]. Incorporating refugee care into local and national health efforts is an important component of multiple Sustainable Development Goals (SDGs) [3].

Displaced persons are increasingly mobile, and humanitarian efforts to support them need to be adapted to this new reality. Mobile populations affected by humanitarian crises who are without access to stable and safe housing face compounded health risks during infectious disease outbreaks, particularly during the novel 2019 coronavirus disease (COVID-19) pandemic. Those living in informal settlements, such as tents and abandoned buildings, are often without access to essential resources like food, soap, and water. In formal settlements, including refugee camps, crowded conditions can increase susceptibility to COVID-19. In these settings, people have been less likely to access medical care or to effectively practice social distancing [4,5].

In Turkey, even prior to the current COVID-19 crisis, Syrian refugees faced many challenges in accessing healthcare. Although Turkey does offer free healthcare to registered refugees in the city within which they are registered through national insurance, mass migration and overstretched health services have complicated efforts to deliver effective care [6]. Multiple barriers to care access exist, including language differences, navigational challenges, lack of medical records, fear of deportation, and xenophobia [7]. Further, refugees experience many conditions that are characteristic of poor social determinants of health: low socioeconomic status, social exclusion, baseline poor nutrition, and unsafe living conditions [8]. Refugee women are considered to be doubly vulnerable, as members of a group that experiences more than one factor diminishing their autonomy. As the study of doubly vulnerable groups presents unique challenges, healthcare needs and concerns of this population are less frequently addressed in the scientific literature [9].

Such vulnerabilities and disparities in access to care and social determinants of health are amplified by COVID-19. For example, rapid information dissemination is particularly challenging for refugee populations that may not speak the majority language of the country they are in. In some settings, the widespread panic was reported to quickly spread through refugee camps due to the lack of information and misinformation among residents who are already “primed for anxiety” [10]. The lack of refugee-specific modalities of care and strategies for information dissemination puts these refugees at risk for poor health outcomes, similar to other vulnerable populations [11].

Team-based care with end-user directed approaches to identify, closely follow up, and support refugee patients has been shown to be an effective model for improving health care in refugee camps [12]. Currently, most healthcare efforts for refugees focus on strengthening the supply side without specifically targeting the demand-side, or individual patient, barriers to access. For example, despite large scale public health efforts and awareness campaigns by the United Nation High Commissioner for Refugees (UNHCR), the Turkish Ministry of Health (MoH), and the World Health Organization (WHO) [4], uptake remains low for preventive healthcare services such as childhood immunizations and antenatal care. Many refugees do not take advantage of these disease prevention interventions [13]. It has been estimated that up to date immunization levels are under 65% and prenatal checkup appointments remain below 30% among the Syrian refugee population in Turkey [14,15].

Timely response strategies remain an urgent priority [16]. The UNHCR continues to strengthen its response to COVID-19 in refugee settlements by establishing Isolation and Treatment Centers (ITCs), scaling hygiene promotion, and reforming water and sanitation delivery. However, as the number of COVID-19 cases among refugee populations continues to grow, collective action and cooperation are required to ensure prompt recognition, treatment, and communication among communities to limit further spread [17]. In this report, we explore the potential for a novel digital health platform to complement these large scale supply-side efforts to improve healthcare access for doubly vulnerable displaced populations.

### Urgent Need for Context-Specific Interventions

Certain precautionary measures have been recommended to limit the spread of COVID-19: hand hygiene, facemasks, social distancing, self-isolation, and adequate nutrition. However, these measures are often not possible for the 5.6 million Syrian refugees in neighboring countries, or displaced persons living under difficult conditions globally. It is important to understand and consider these unique needs in both the planning and execution of a comprehensive COVID-19 response, with particular attention to preparedness, surveillance, and communication plans. Preventing infection is the best way to protect both refugee and host communities; however, there is little evidence on how to best do this for those affected by a humanitarian crisis, during a global pandemic from an emerging disease. Directed research on agile interventions is crucial to better understand target areas for scale during rapidly evolving crises.

Unlike in prior epidemics, the overwhelming majority of reported deaths from COVID-19 have been in higher income countries (HICs) [17]. While these countries appear to have been affected by COVID-19 earlier due to social mobility, the virus quickly spread worldwide to include outbreaks in low- and middle-income countries (LMICs). While HICs have struggled to manage the pandemic, LMICs face potentially devastating morbidity and mortality, given their relative lack of resources and context-specific interventions [18]. Demographic differences across these contexts can be significant enough to affect population-level behavior and epidemiology. For example, the population of Syrian refugees in and around Istanbul alone is estimated to be one million, with 43% under 18, and 47% female; this is younger than the local population in the same regions [2]. Thus, a mobile health application targeted towards a younger and female demographic could provide an important modality for public health interventions.

### Uptake and Sustainability of Mobile Technology: Solutions in the Field

Information and communication technology infrastructure has grown substantially over the past two decades worldwide, and particularly in LMICs over recent years [18]. Communicating and collecting data with innovative and user-friendly mobile technologies could serve as an important tool for accessing displaced and other highly mobile populations. Such a tool could also serve to empower this population by increasing control over their own health.

Evidence on the sustainability of mhealth implementation in traditionally resource-constrained environments is limited but continues to grow [19,20,21,22,23]. Positive long-term impact of mHealth in LMICs has been demonstrated with chronic disease management [24], including behavior change [25], and medication adherence [26]. In particular, mobile technology has been an effective tool to reduce the burden of disease through more efficient prevention, treatment, education, data collection, and management support in HIV/AIDS and tuberculosis care [24]. In complex humanitarian emergencies, improved surveillance and monitoring with mHealth applications has been shown, with examples including the West African Ebola outbreak, the Haitian earthquake, and the cholera outbreak [27,28,29]. While successful mhealth innovations exist in humanitarian contexts globally, quality evidence is overall limited and requires ongoing contextualization of local needs.

The HERA App is an open-source mobile application specifically designed for the Syrian refugee population in Turkey [30]. It was created in 2018 to harness the high levels of smartphone usage among the Syrian refugee community in Turkey to improve access to healthcare. The mobile application enables users to safely and privately receive healthcare appointment reminders, access health-related communication, store medical records, contact emergency services, and navigate the Turkish healthcare system in the three most commonly spoken local languages: Arabic, Turkish, and English [30]. A feasibility pilot study of 200 Syrian refugee women in Istanbul who were either pregnant or had at least one child under the age of two was conducted in 2018 with the HERA App. It found that automated reminders for antenatal visits and childhood immunizations were effective in improving compliance, were positively received, and can be a low-cost, high-value alternative to other reminder methods [31,32]. Importantly, the study confirmed that these women had access to smartphones and that they use them as a method of accessing health information.

In response to the evolving COVID-19 pandemic, the HERA App was modified to include a COVID-19 response component in March of 2020. HERA App was chosen as it was already in use and shown to be a feasible method of mobile communication by the target population. The education content was adapted to include general information about COVID-19, including basic protective measures, a virus tracking map, government restrictions, and testing site referrals. Users were notified about these updates on their mobile devices. This intervention was performed in a primarily urban setting with Syrian refugees who live in and around Istanbul, Turkey, focusing on women, under a previously obtained ethics review board approval from Acibadem University. Following the initial incorporation of these educational features to the HERA App, 75% (n = 150) of the user-base was successfully contacted for symptomatic assessment at two-week intervals.

### The Future of mHealth in the Refugee Context

Novel mHealth applications can enable broad use of innovative end-user centered interventions for highly mobile populations during a pandemic response. Within this context, they can be used to: 1) widely disseminate health information for refugees, 2) identify and educate high-risk mobile populations to ensure proactive screening and early case identification, 3) support data collection of mobile populations during infectious outbreaks, and 4) decompress hospital-based triage by improving access to health information and safety planning.

MHealth tools can be a low-cost and agile method to rapidly adapt and scale both individual and population-level health interventions. In our example, uptake of the HERA App in Turkey was rapid, and users received education on preventing the spread of COVID-19 while completing regular COVID-19 symptomatic self-assessments. This approach can be broadened to include additional health interventions, including increased education, contact-tracing, and triage support for overstretched public services. As infectious disease outbreaks exacerbate pre-existing health disparities, particularly among maternal, child, and refugee health, mHealth can be used to increase access to education and outreach for these doubly vulnerable populations [33]. In lower-resourced regions with poor access to health care, where access to a cell phone is often easier than soap, mHealth can play an important role in beginning to close the disparity gap [34]. Mobile health platforms can serve as a key policy innovation for future outbreaks and other urgent global needs for diverse and moving populations in humanitarian crises.

This mHealth intervention is uniquely designed to provide focused behavioral influence to increase the uptake of available public health services. The platform utilized an agile methodology framework with an iterative improvement process, allowing for meaningful incorporation of user-identified feedback on application features. Importantly, it was initially designed not only as a medical tool but also to facilitate female empowerment and to encourage independent learning. This targeted education promotes the involvement of Syrian women in the decision-making process about the family’s health. Building capacity for health literacy within a population historically susceptible to poverty and gender inequality is a stepping-stone in achieving health as a human right [35].

MHealth interventions propose a unique, value-based opportunity to communicate with moving populations, as users do not need to carry anything other than a phone, and may provide affordable alternatives for information dissemination and health behavior change. When scaled, interventions such as this may allow for accurate symptomatic tracking, health education outreach, and triage offloading with minimal personnel and financial resource input, in order to reduce exposure risks within the health system. The data collection capacity allows for timely policy responses and subsequent cost-effective interventions for mobile populations.

### Limitations

This was an exploratory evaluation of a modification to an mHealth platform, and the findings are limited by small sample size and lack of a control group. Baseline data for population size, clinic appointment compliance, and vaccination status is difficult to assess, given the high mobility of the population. Only self-assessment of the presence or absence of COVID-19 symptoms was performed, and COVID-19 infection incidence by laboratory testing among participants was not assessed.

## Conclusion

Disparities in access to health care and health information by refugees are heightened during infectious disease outbreaks. Mobile technology can play an important role in accessing and communicating with otherwise difficult to reach highly mobile populations, such as Syrian refugees in Turkey. The HERA App is an example of an mHealth platform that can be rapidly distributed at low cost to improve access to care and information dissemination. Additionally, similar mHealth applications can be rapidly adapted to emerging challenges, including the COVID-19 pandemic. Future research should evaluate the feasibility of mHealth platforms such as this in similar high-risk and difficult to reach populations, as well as evaluate their impact on targeted health behavior change and patient-centered outcomes.
